# Macitentan for the Treatment of Refractory Digital Ulcers in Patients With Connective Tissue Diseases

**DOI:** 10.7759/cureus.38303

**Published:** 2023-04-29

**Authors:** Clara Soto Abánades, Ana Noblejas Mozo, Gema Bonilla Hernán, Jorge Alvarez Troncoso, Juan José Ríos Blanco

**Affiliations:** 1 Internal Medicine, Systemic Autoimmune Diseases Unit, Hospital Universitario La Paz, Madrid, ESP; 2 Rheumatology, Systemic Autoimmune Diseases Unit, Hospital Universitario La Paz, Madrid, ESP

**Keywords:** scleroderma, systemic sclerosis, digital ulcers, macitentan, bosentan, endothelin receptor antagonists, connective tissue disease, pulmonary arterial hypertension, vasodilator therapy, prostaglandin therapy

## Abstract

Systemic sclerosis (SSc) is a chronic autoimmune disease with complex pathogenesis, characterized by vascular dysfunction and fibrosis. Digital ulcers (DUs) are a common and severe complication in SSc patients, negatively impacting their quality of life. This retrospective study evaluates the use of macitentan, an endothelin receptor antagonist, in six female patients with connective tissue disease (CTD) and sclerodermiform features (five SSc and one mixed connective tissue disease) for the treatment of refractory DUs. Macitentan demonstrated a safe and effective alternative to bosentan, reducing DU relapses, hospitalizations, and the use of systemic prostaglandin therapy. The findings suggest that macitentan may be a valuable therapeutic option in specific cases of recurrent or refractory DUs and warrant further investigation in larger, long-term studies.

## Introduction

Systemic sclerosis (SSc) or scleroderma is a chronic autoimmune disease characterized by generalized vascular dysfunction and progressive fibrosis of the skin and internal organs [1]. SSc presents with a diverse range of manifestations that may affect various parts of the body, including skin thickening, Raynaud's phenomenon, gastrointestinal symptoms, interstitial lung disease (ILD), pulmonary arterial hypertension (PAH), and arthritis, among others [1-3]. It is classified into two main clinical subtypes based on the extent of skin involvement: limited cutaneous systemic sclerosis (lcSSc) and diffuse cutaneous systemic sclerosis (dcSSc) [1-3]. The pathogenesis of SSc is complex and not entirely clear. It is postulated that immune activation, vascular damage, and excessive extracellular matrix production are the most important factors in disease development [2,3].

Digital ulcers (DUs) are a frequent and serious complication in SSc. They appear in up to 50% of patients at some point, and their occurrence is more frequent in dcSSc. Their presence significantly worsens the quality of life of patients and darkens their prognosis as they present more vascular complications due to common pathophysiological mechanisms [4,5].

Calcium channel blockers (CCBs) such as amlodipine, nifedipine, and diltiazem are used to treat DUs in SSc as first-line systemic pharmacologic treatment besides supportive means like topical ointments/creams [6]. In cases of lack of efficacy or contraindications of these drugs, the use of phosphodiesterase 5 inhibitors (PDE5) (sildenafil and tadalafil) and endothelin receptor antagonists (ERAs) is advised; both classes of vasodilators are indicated in PAH. Other treatments for DUs include topical nitrates, angiotensin-converting enzyme inhibitors (ACEIs), angiotensin II receptor blockers (ARBs), statins, and antiplatelet drugs. Refractory or more severe cases respond to systemic prostaglandins [6-10].

Currently, bosentan is the only ERA authorized in Europe for the prevention of the recurrence of DUs in patients with SSc [11]. This recommendation is based on two clinical trials that demonstrated the efficacy of bosentan for this indication [12,13]. Bosentan has also been shown to improve the course of existing ulcers [14,15]. Macitentan is a newer generation ERA that achieves more sustained receptor binding and superior tissue penetration compared to other drugs in the same family [16,17]. However, two clinical trials did not reach statistical significance in terms of efficacy in preventing new DUs, although there are case reports that have demonstrated its efficacy, even in some of the patients included in the DUAL-2 trial [18-20]. In clinical practice, treatment with macitentan for DUs has achieved excellent course evolution as well as prevention of recurrences [19].

Our objectives were to describe the demographic, clinical, and immunological characteristics of a series of patients with SSc (or CTD with overlap features of SSc) treated with macitentan for DUs and to evaluate the treatment, evolution, and recurrence of DUs before and after macitentan administration.

## Materials and methods

Study design and population

This retrospective, observational study was conducted at a single center, involving patients with systemic sclerosis (SSc) or connective tissue disease (CTD) with overlap features of SSc treated with macitentan for DUs. We reviewed the medical records of these patients between January 2017 and December 2022. Inclusion criteria were (1) a diagnosis of SSc or CTD with overlap features of SSc (skin thickening, DUs, Raynaud's phenomenon, and pathological capillaroscopy), (2) presence of DUs, and (3) treatment with macitentan. Exclusion criteria were (1) patients under 18 years of age, (2) pregnant or lactating women, and (3) patients with contraindications to macitentan therapy.

Data collection

Data were collected from electronic medical records and included demographic, clinical, and immunological characteristics as well as treatment history, evolution, and recurrence of DUs before and after macitentan administration. Demographic data included age at the start of macitentan treatment and age at disease onset. Clinical data encompassed SSc subtype (lcSSc or dcSSc), Raynaud's phenomenon, skin involvement, ILD, PAH, gastroesophageal involvement, and comorbidities. Immunological data included autoantibodies associated with SSc or CTD. Treatment data consisted of previous and concomitant medications for DUs and other SSc-related manifestations. DU evolution and recurrence were assessed based on clinical notes and available imaging studies.

Statistical analysis

Descriptive statistics were used to analyze the demographic, clinical, and immunological characteristics of the study population. Continuous variables were expressed as means and ranges, while categorical variables were presented as counts and percentages. The treatment, evolution, and recurrence of DUs before and after macitentan administration were described using counts and percentages, and comparisons were made using the McNemar test for paired data. All statistical analyses were performed using Wizard Pro for Mac version 2.0.13 (262).

Ethical considerations

As the study was retrospective and observational, informed consent was waived. All patient data were anonymized and maintained confidentially in accordance with the Declaration of Helsinki and relevant local regulations.

## Results

A retrospective study was conducted to evaluate the use of macitentan for treating DU. The study included six female patients with a mean age of 61.50 years (range: 39-75) at the start of macitentan treatment and a mean age of 50.93 years (range: 21-77) at the onset of the disease. Of the six patients, five had SSc (three had dcSSc, and two had lcSSc), and one patient had mixed connective tissue disease (MCTD) with sclerodermiform features.

All patients had skin thickening, Raynaud's phenomenon, positive antinuclear antibodies (ANA), and pathologic capillaroscopy (late sclerodermiform pattern). Four patients had ILD (Table 1). Two patients had PAH, and five had concomitant gastroesophageal involvement. In terms of comorbidities, two patients had type 2 diabetes mellitus, two had hypertension, one had dyslipidemia, one had atrial fibrillation, one had autoimmune hypothyroidism, one had ischemic heart disease, and one had valvulopathy (mitral stenosis).

**Table 1:** Baseline demographic, clinical, and immunological characteristics of the study population AAV: ANCA-associated vasculitis; ANA: Antinuclear antibodies; aPLs: Antiphospholipid antibodies; APS: Antiphospholipid syndrome; CEN-B: Anti centromere antibodies; CNS: Central nervous system; dcSSc: Diffuse cutaneous systemic sclerosis; lcSSc: Limited cutaneous systemic sclerosis; MCTD: Mixed connective tissue disorder; PBC: Primary biliary cholangitis; RF: Rheumatoid factor.

Patient	#1	#2	#3	#4	#5	#6
Age at onset (years)	50	52	51	48	77	21
Age at evaluation (years)	59	57	68	71	75	39
Smoking status	No	No	No	No	No	Previous
Connective tissue disorder	dcSSc, AAV	dcSSc	lcSSc	dcSSc	lcSSc	MCTD, APS
Autoantibodies	ANA, Scl-70, MPO (p-ANCA), RF	ANA, Scl-70	ANA, CEN-B	ANA,Scl-70, Ro/SSA	ANA, CEN-B	ANA, RNP, Ro/SSA, dsDNA, Sm, aPLs
Interstitial lung disease	Yes	Yes	No	Yes	No	Yes
Pulmonary arterial hypertension	No	No	Yes	Yes	No	No
Gastroesophageal involvement	Yes	Yes	No	Yes	Yes	Yes
Other involvements	-	Calcinosis	PBC, intestinal	CNS, articular	-	Myositis, serositis, enteritis

Regarding the treatment of Raynaud's phenomenon and DU (Table 2), five patients were receiving ACEI/ARB, three with CCB (the remaining patients could not tolerate it due to hypotension), five with statins, four with antiplatelets, and one with rivaroxaban (switched from aspirin after the onset of atrial fibrillation). Three patients were treated with PDE5 inhibitors. Prior to the start of macitentan, all patients had been treated with ERA (bosentan). The average time of use of bosentan until the switch to macitentan was 57.60 months (range: 0.60-123.36). The average treatment time with macitentan was 45.99 months (range: 26.04-60.00).

**Table 2:** Treatment of digital ulcers ACEI: Angiotensin-converting enzyme inhibitors; ARB: Angiotensin receptor blockers; CCB: Calcium channel blockers; DMARD: Disease-modifying antirheumatic drugs; ERA: Endothelin receptor antagonists; HCQ: Hydroxychloroquine; PDE5: Phosphodiesterase-5; SP: Systemic prostaglandin.

Patient	#1	#2	#3	#4	#5	#6
ACEI/ARB	Yes	No	Yes	Yes	Yes	Yes
CCB	Yes	Yes	No	No	Yes	No
Statin	Yes	Yes	Yes	Yes	Yes	No
Antiplatelets/anticoagulation	Clopidogrel	Aspirin	Aspirin	Rivaroxaban	-	Aspirin
ERA	Yes	Yes	Yes	Yes	Yes	Yes
PDE5 inhibitors	-	Sildenafil	Sildenafil	Tadalafil	-	-
DMARD	Mycophenolate	Tacrolimus; rituximab	-	Mycophenolate	HCQ; colchicine	HCQ; tacrolimus; colchicine; tocilizumab
Cycles of SP before macitentan	3	4	2	0	1	2
Cycles of SP after macitentan	0	0	1	0	1	0
Bosentan time (months)	58.44	55.08	123.36	12.12	0.60	97.44
Macitentan time (months)	26.04	60.00	45.00	45.96	30.96	60.00

Overall, the reasons for initiating macitentan treatment in this study were unfavorable evolution despite bosentan (two cases), the need to optimize vasodilator therapy for PAH (two cases), hepatotoxicity (one case), and interaction between bosentan and tacrolimus (one case).

Five patients had previously required treatment with systemic prostaglandins (alprostadil) for refractory DU. The average number of alprostadil cycles prior to the start of macitentan was 2.0 (range: 0-4), and the average number of cycles after its initiation was 0.33 (range: 0-1). Macitentan treatment resulted in an 83.5% reduction in the number of systemic prostaglandin cycles.

Of the six patients included, two (#1 and #2) received macitentan through compassionate use after experiencing complications with DU from previous treatments with alprostadil, nifedipine, sildenafil, and bosentan. After switching to macitentan, both patients did not present new ulcers.

Two other patients (#3 and #4) received macitentan to treat the progression of PAH associated with SSc after previous treatments with bosentan and sildenafil/tadalafil. One patient had previously experienced severe events with digital necrosis and amputation before the ERA change, while the other had only presented with a single complicated ulcer that resolved with local care. After receiving macitentan, neither patient presented new episodes of DU.

**Figure 1:**
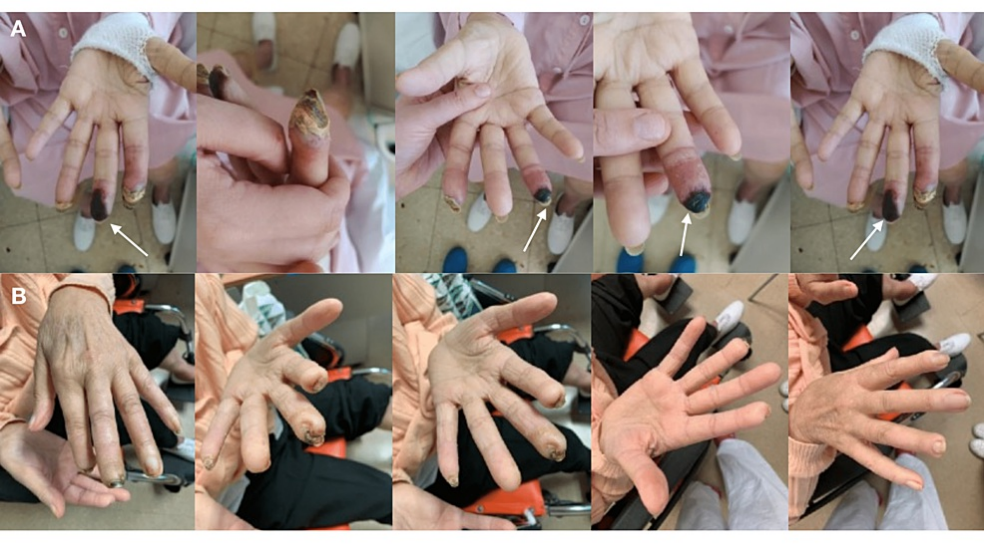
Digital ulcers on patient #5 before and after treatment with macitentan Digital ulcers on patient #5 before (A) and after (B) treatment with macitentan. Necrotic areas (white arrow pointing to the third finger of the left hand) and scarring areas are shown in the top row (A), and a very favorable evolution, several weeks after switching from bosentan to macitentan, is shown in the bottom row.

For patient #5, macitentan was added due to complications with hypertransaminasemia while using bosentan after the first episode of a DU. After rapidly switching to macitentan, the patient's transaminase levels normalized, the previous DU resolved (Figure 1), and no new DU appeared.

Macitentan was initiated in patient #6 due to an interaction between bosentan and tacrolimus. Two years later, the patient had a single episode of DU that did not require hospitalization or systemic prostaglandin therapy.

None of the patients experienced clinical or analytical adverse effects after the introduction of macitentan. All the patients showed improvement in the number, duration, and appearance of new DU as well as in the need for systemic prostaglandins.

## Discussion

In our cohort of six patients with complex CTD with sclerodermiform features (five SSc and one MCTD), we observed a lower number of DU relapses after macitentan treatment. Macitentan was well-tolerated and reduced systemic prostaglandin use by 83.5%.

Bosentan has been shown to be effective in treating and preventing DU [11-13]. However, macitentan did not meet the primary outcome (the cumulative number of new DUs from baseline to week 16) in clinical trials (DUAL-1 and DUAL-2) [18]. Patients on PDE5 inhibitors and prostaglandin therapy in the three months prior to screening and those with comorbidities affecting hand functionality or who were smokers were excluded from the macitentan trials [18-20]. Patients were randomized to receive placebo, macitentan 3 mg, or macitentan 10 mg (the only dose marketed after the SERAPHIN pivotal trial in PAH [20]). Although the expected result was not achieved, the trial was short-term compared to the real-life data presented in our series. Furthermore, patients with other DU treatments were excluded, so more severe cases were not recruited. Perhaps the short duration of clinical trials, earlier diagnosis of SSc, better and faster treatment, and improved standard treatment of DU may have influenced a lower number of DU and less margin for improvement. These trials included patients with SSc and a previous history of recurrent DU as well as at least one active new-onset or worsening DU in the eight weeks before screening. Despite being a more potent ERA than bosentan, it is unclear why macitentan did not achieve the expected results in relation to DU.

It is essential to note that all patients in our cohort received classic and biologic immunosuppressive treatment, which may have influenced the treatment response, as DUs have inflammatory, vascular, and fibrotic components that may respond to immunosuppressive therapy. However, as there are only six patients, it is impossible to draw reliable conclusions, but rather new questions and hypotheses are proposed.

In our cohort, macitentan has been an effective and safe alternative for preventing relapses of DU in patients with complex CTD with sclerodermiform features and for avoiding the need for hospitalization or the use of systemic prostaglandin therapy. We, therefore, consider that macitentan may be a good therapeutic option in specific cases with recurrent or refractory DU. Additionally, macitentan should be considered in SSc-DU if contraindicated with bosentan due to toxicity, interactions, or indication of macitentan due to PAH. Given the absence of a clinical trial whose results support the use of macitentan for the prevention of DUs in SSc, further studies with other designs and longer duration would be beneficial to better understand the role of macitentan in treating DU and determining the possible influence of immunosuppressive treatments on patient outcomes.

## Conclusions

Systemic sclerosis (SSc) is a complex autoimmune disease characterized by diverse manifestations and complications, including DUs. While bosentan has been shown to be effective in treating and preventing DUs, macitentan, a newer generation ERA, did not meet the primary outcome in clinical trials. However, our retrospective study suggests that macitentan may be an effective and safe alternative for preventing DU relapses in patients with complex CTD with sclerodermiform features. Macitentan has the potential to reduce the need for hospitalization and systemic prostaglandin therapy in these patients. Our findings indicate that macitentan may be a valuable therapeutic option in specific cases with recurrent or refractory DU as well as in SSc-DU cases where bosentan is contraindicated. Given the absence of clinical trials supporting the use of macitentan for DU prevention in SSc, further studies with alternative designs and longer durations are warranted to confirm and expand upon these findings.

## References

[1] Denton CP, Khanna D (2017). Systemic sclerosis. Lancet.

[2] Di Benedetto P, Ruscitti P, Liakouli V, Cipriani P, Giacomelli R (2019). The vessels contribute to fibrosis in systemic sclerosis. Isr Med Assoc J.

[3] Pope JE, Denton CP, Johnson SR, Fernandez-Codina A, Hudson M, Nevskaya T (2023). State-of-the-art evidence in the treatment of systemic sclerosis. Nat Rev Rheumatol.

[4] Denton CP, Krieg T, Guillevin L (2012). Demographic, clinical and antibody characteristics of patients with digital ulcers in systemic sclerosis: data from the DUO Registry. Ann Rheum Dis.

[5] Mihai C, Landewé R, van der Heijde D (2016). Digital ulcers predict a worse disease course in patients with systemic sclerosis. Ann Rheum Dis.

[6] Zidek W, Spiecker C, Knaup G, Steindl L, Breuer HW (1995). Comparison of the efficacy and safety of nifedipine coat-core versus amlodipine in the treatment of patients with mild-to-moderate essential hypertension. Hypertension Study Group. Clin Ther.

[7] Sagonas I, Daoussis D (2023). Treatment of digital ulcers in systemic sclerosis: recent developments and future perspectives [IN PRESS]. Clin Rheumatol.

[8] Wigley FM, Seibold JR, Wise RA, McCloskey DA, Dole WP (1992). Intravenous iloprost treatment of Raynaud's phenomenon and ischemic ulcers secondary to systemic sclerosis. J Rheumatol.

[9] Badesch DB, Tapson VF, McGoon MD (2000). Continuous intravenous epoprostenol for pulmonary hypertension due to the scleroderma spectrum of disease. A randomized, controlled trial. Ann Intern Med.

[10] Scorza R, Caronni M, Mascagni B (2001). Effects of long-term cyclic iloprost therapy in systemic sclerosis with Raynaud's phenomenon. A randomized, controlled study. Clin Exp Rheumatol.

[11] Jerjen R, Nikpour M, Krieg T, Denton CP, Saracino AM (2022). Systemic sclerosis in adults. Part II: management and therapeutics. J Am Acad Dermatol.

[12] Korn JH, Mayes M, Cerinic MM (2004). Digital ulcers in systemic sclerosis: prevention by treatment with bosentan, an oral endothelin receptor antagonist. Arthritis Rheum.

[13] Matucci-Cerinic M, Denton CP, Furst DE (2011). Bosentan treatment of digital ulcers related to systemic sclerosis: results from the RAPIDS-2 randomised, double-blind, placebo-controlled trial. Ann Rheum Dis.

[14] Ivorra JAR, Simeon CP, Sancho JJA, Egurbide MV, Castillo MJ, Lloria X, Fonollosa V (2011). Bosentan in clinical practice for treating digital and other ischemic ulcers in Spanish patients with systemic sclerosis: IBER-DU cohort study. J Rheumatol.

[15] Tsifetaki N, Botzoris V, Alamanos Y, Argyriou E, Zioga A, Drosos AA (2009). Bosentan for digital ulcers in patients with systemic sclerosis: a prospective 3-year followup study. J Rheumatol.

[16] Sidharta PN, van Giersbergen PL, Dingemanse J (2013). Safety, tolerability, pharmacokinetics, and pharmacodynamics of macitentan, an endothelin receptor antagonist, in an ascending multiple-dose study in healthy subjects. J Clin Pharmacol.

[17] Gatfield J, Grandjean CM, Sasse T, Clozel M, Nayler O (2012). Slow receptor dissociation kinetics differentiate macitentan from other endothelin receptor antagonists in pulmonary arterial smooth muscle cells. PLoS One.

[18] Khanna D, Denton CP, Merkel PA (2016). Effect of macitentan on the development of new ischemic digital ulcers in patients with systemic sclerosis: DUAL-1 and DUAL-2 randomized clinical trials. JAMA.

[19] Gonçalves T, Santos L (2019). Macitentan in the treatment of severe digital ulcers. BMJ Case Rep.

[20] Pulido T, Adzerikho I, Channick RN (2013). Macitentan and morbidity and mortality in pulmonary arterial hypertension. N Engl J Med.

